# Proximal tibial tuberculous osteomyelitis with secondary knee joint arthritis: A case report

**DOI:** 10.1016/j.radcr.2025.12.019

**Published:** 2026-01-15

**Authors:** Ilyesse Haichour, Kamal Benalia, Najib Abdeljaouad, Hicham Yacoubi

**Affiliations:** a Faculty of Medicine and Pharmacy, Mohammed Ist University, Oujda, Morocco; b Department of Traumatology, Orthopedic Mohammed VI University Hospital Mohammed I University, Oujda, Morocco; c Faculty of Medicine and Pharmacy, Mohammed First University, LAMCESM, Oujda, Morocco

**Keywords:** Proximal tibia, Osteolytic lesion, Tuberculosis, Imaging, Biopsy, Osteomyelitis, Tuberculous arthritis

## Abstract

Tuberculous osteomyelitis is an uncommon form of musculoskeletal tuberculosis and most frequently involves long bones such as the femur and tibia. We report the case of a 53-year-old woman presenting with a one-year history of progressive pain and swelling of the left knee. Imaging revealed a well-defined lytic cavitary lesion in the metaphyseal–epiphyseal region of the proximal tibia with cortical breach, intra-articular extension, and direct communication with a pre-tibial soft-tissue abscess. Surgical management included debridement and curettage of the intramedullary cavity, drainage of the pre-tibial collection, PMMA cement filling, and a knee joint washout. Histopathological analysis demonstrated epithelioid granulomas with Langhans-type giant cells and caseous necrosis, and PCR confirmed *Mycobacterium tuberculosis*. This case highlights the importance of considering tuberculous osteomyelitis in the differential diagnosis of chronic osteolytic lesions with associated soft-tissue collections or secondary joint involvement, particularly in endemic regions.

## Introduction

Tuberculosis remains a significant global health concern, with extrapulmonary forms accounting for up to 20% of cases [1]. Among these, osteoarticular tuberculosis represents a small but impactful subset, most frequently affecting the spine and large weight-bearing joints [1,2]. In contrast, involvement of long bones—particularly the proximal tibia—is uncommon and often leads to diagnostic delay because of its nonspecific clinical and radiologic presentation [3].

Secondary extension from metaphyseal bone into the knee joint may result in chronic monoarthritis, mimicking pyogenic infection, inflammatory arthropathies, or even neoplastic disease. Recognizing these atypical presentations is essential for timely diagnosis and appropriate management.

We report a rare case of proximal tibial tuberculous osteomyelitis with secondary knee joint arthritis, highlighting the key diagnostic features and therapeutic approach.

## Case report

**Fig. 1:**
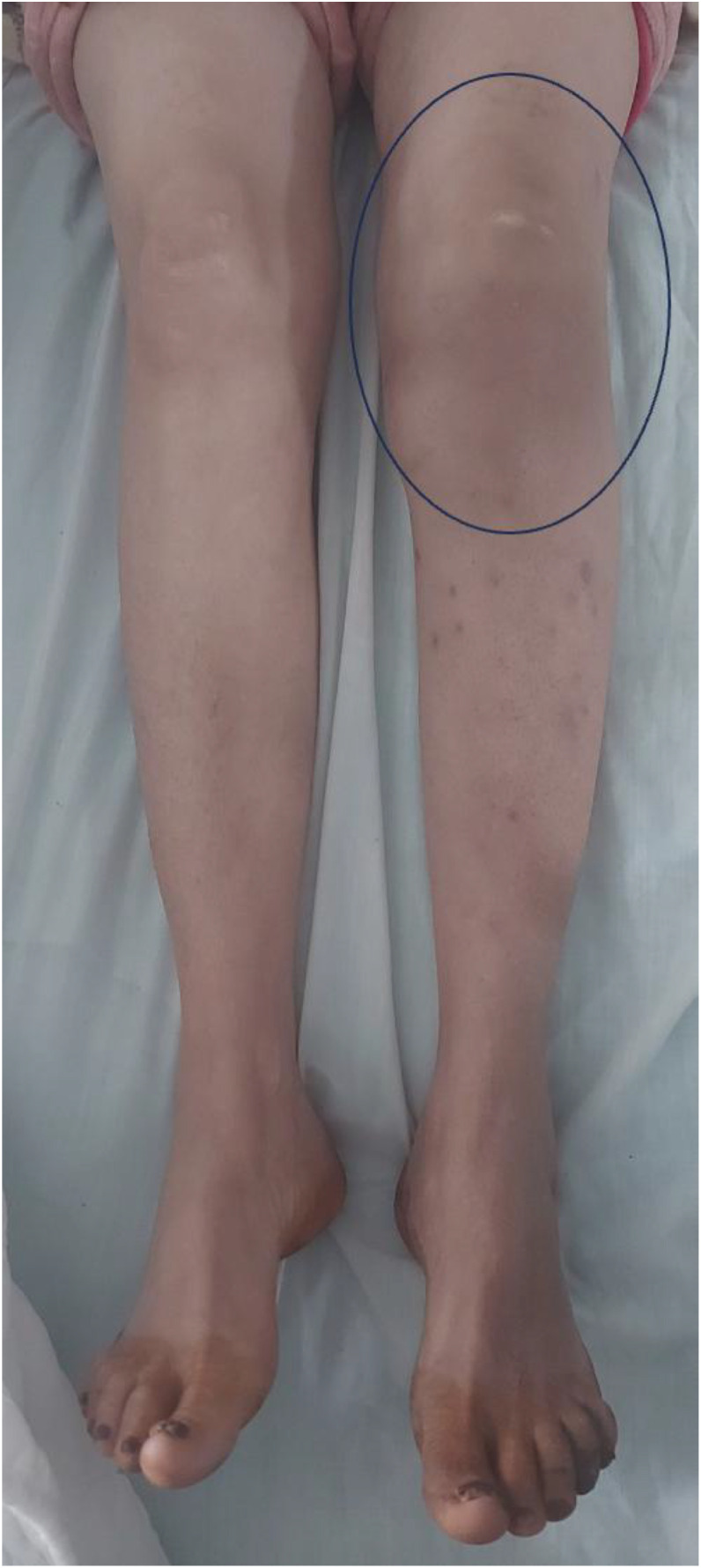
Clinical image showing swelling and erythema around the left knee of the patient.

We report the case of a 53-year-old woman with no significant past medical history, vaccinated with BCG during childhood, who presented with a progressively enlarging swelling over the anteromedial aspect of the left knee (Fig. 1).

The symptoms had begun approximately one year earlier with intermittent inflammatory pain partially relieved by NSAIDs, later progressing to persistent swelling, general fatigue, and profuse night sweats.

Clinical examination revealed localized erythema and a fluctuant swelling over the anteromedial proximal tibia, with a preserved flexion-extension range of motion and a positive ice-pack test. There were no clinical signs of pulmonary or extrapulmonary tuberculosis elsewhere.

Laboratory investigations demonstrated nonspecific inflammatory findings, including CRP at 52 mg/L (normal < 5 mg/L), ESR at 34 mm/h (normal < 20 mm/h), white blood cell count of 7.8 × 10⁹/L (normal 4-10 × 10⁹/L), hemoglobin of 12.6 g/dL (normal 12-16 g/dL), and platelet count of 315 × 10⁹/L (normal 150-400 × 10⁹/L).

Joint aspiration yielded inflammatory synovial fluid with low cellularity (3800 neutrophils/mm³), and cultures were sterile.

**Fig. 2:**
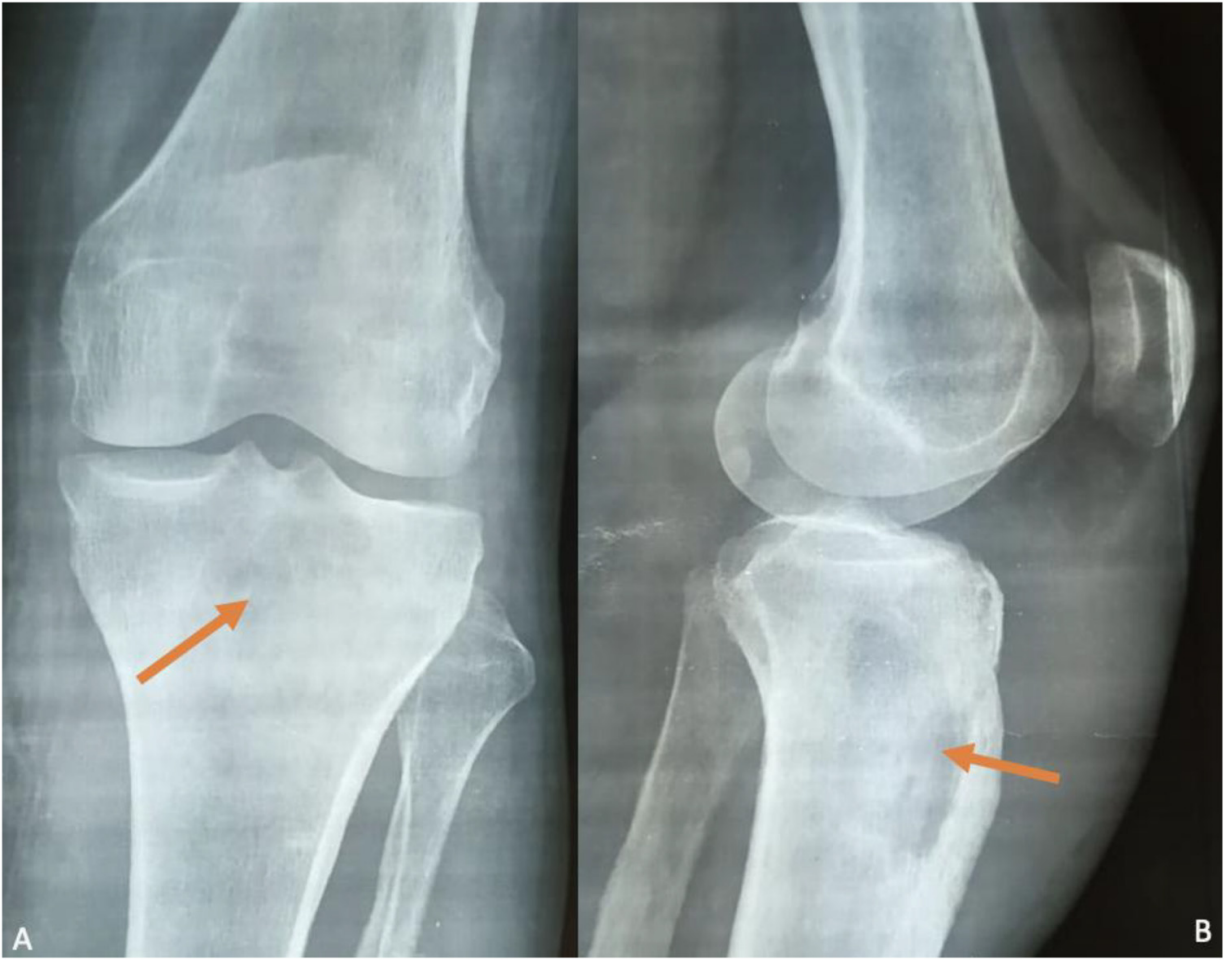
Plain radiographs of the knee, anteroposterior (A) and lateral views (B), show a well-defined pathological metaphyseal tibial lytic lesion (orange arrow) associated with marked peripheral sclerotic reaction.

Plain radiographs showed a pathological metaphyseal tibial lytic lesion associated with marked peripheral sclerotic reaction and mild joint space narrowing (Fig. 2)*.* Chest radiograph was unremarkable.

**Fig. 3:**
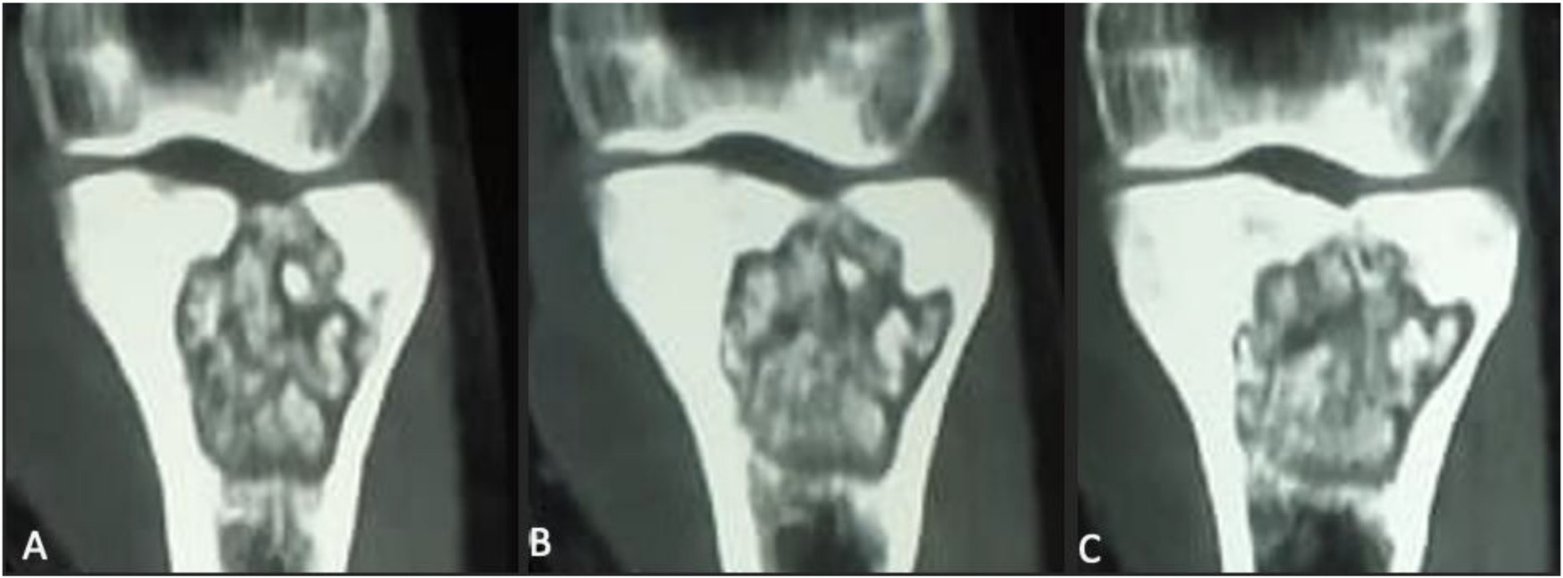
Coronal CT scans without contrast injection of the knee show a well-defined cavitary lytic lesion (C) in the metaphyseal-epiphyseal region of the tibia, with heterogeneous content, cortical bone lysis, and intra-articular extension into the knee joint (A and B).

**Fig. 4:**
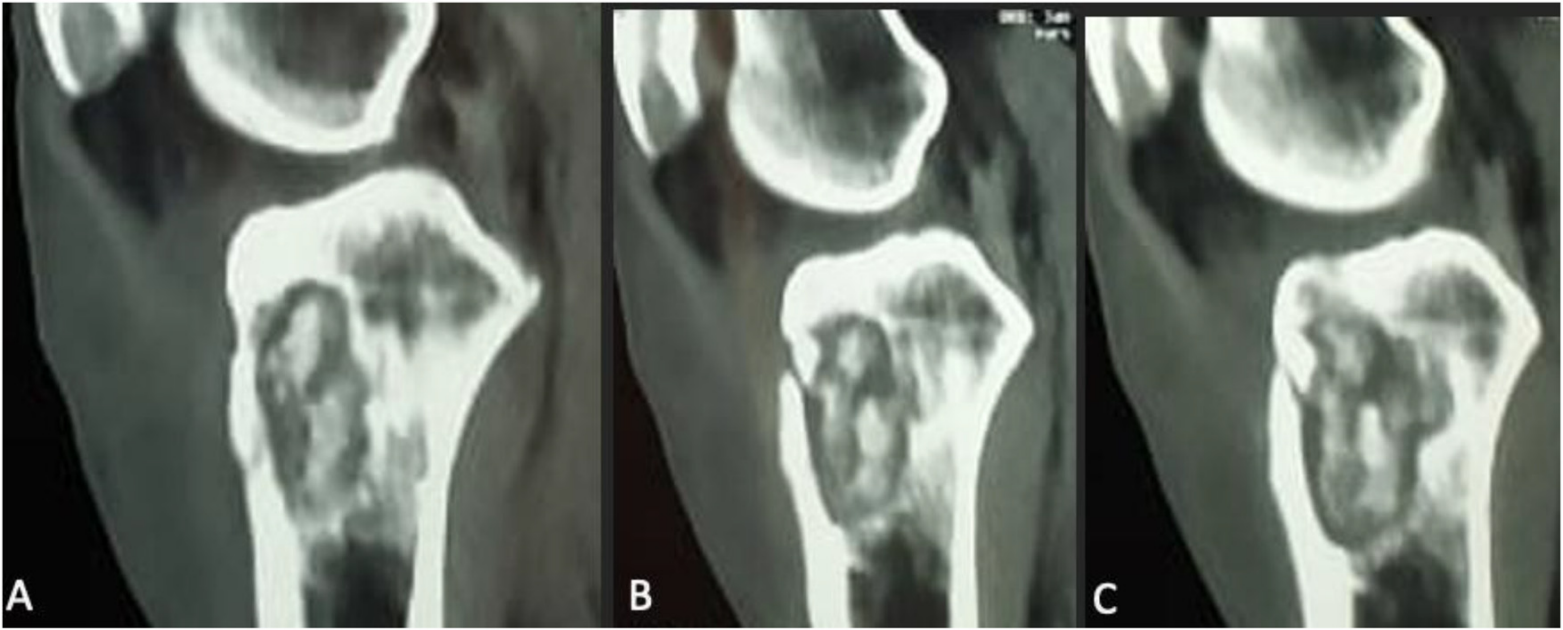
Sagittal CT scans without contrast injection of the knee show a well-defined cavitary lytic lesion (A) in the metaphyseal-epiphyseal region of the tibia, with heterogeneous content and disruption of the anterior cortex consistent with a pathologic fracture (B and C).

**Fig. 5:**
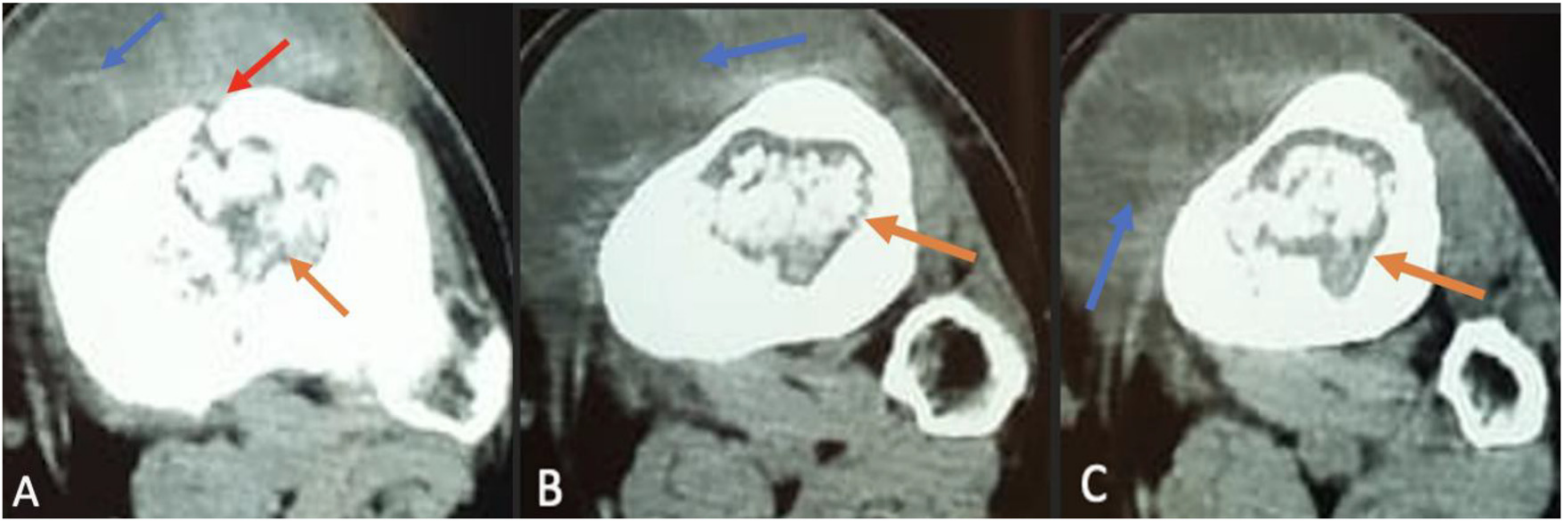
Axial CT scans without contrast injection show a well-defined lytic lesion (orange arrow) in the proximal left tibia, with anterior cortical breach (red arrow) and adjacent cortical destruction, communicating with a hypodense pre-tibial soft-tissue collection (blue arrow). A surrounding rim of intramedullary sclerosis is also visible, consistent with tuberculous osteomyelitis with associated soft-tissue abscess.

CT imaging without contrast injection demonstrated a well-defined lytic cavitary lesion of the proximal tibia with an intra-articular cortical breach. Anterior cortical destruction was clearly visible, with direct communication between the intramedullary cavity and the pre-tibial hypodense soft-tissue collection, supporting a pattern of contiguous spread rather than isolated hematogenous involvement (Fig. 3, Fig. 4, Fig. 5)*.*

**Fig. 6:**
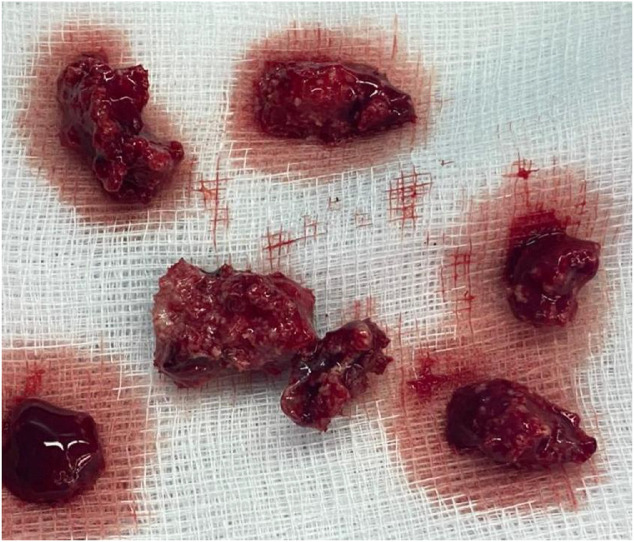
Intraoperative image showing the resection of necrotic tissues and osseous sequestra, which were sent for histopathological examination.

**Fig. 7:**
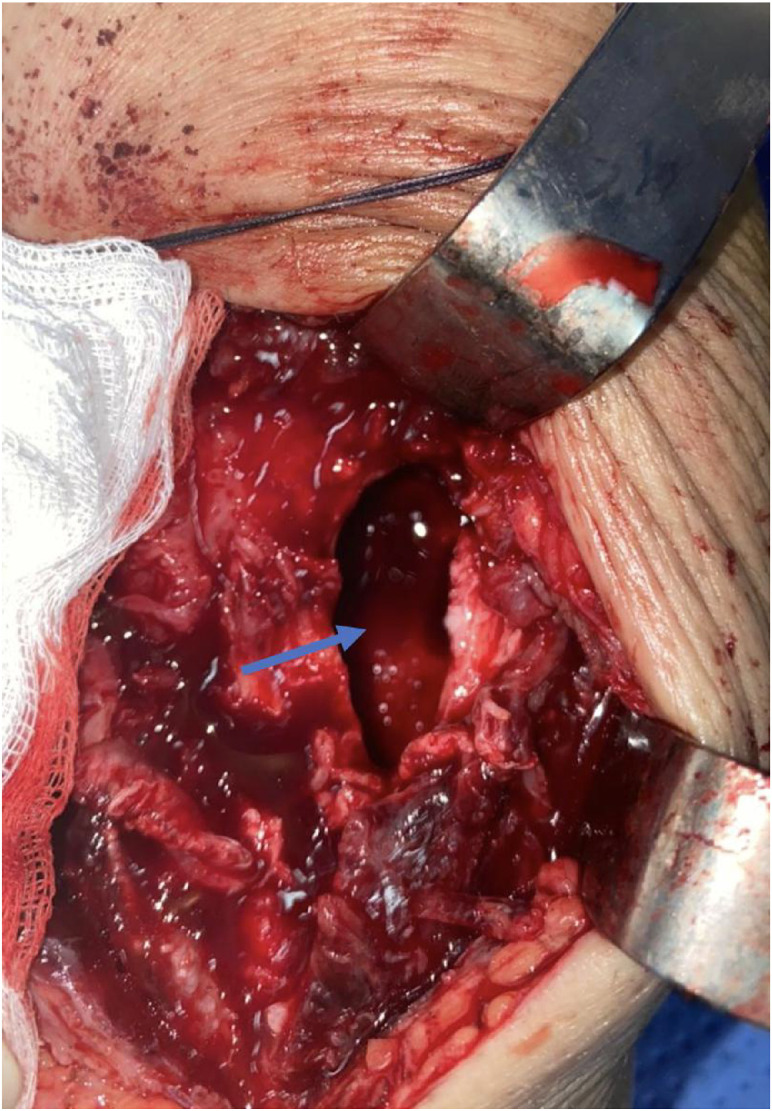
Intraoperative image showing the cavitary lesion (blue arrow) at the level of the tibial metaphysis after debridement and resection of suspicious tissues.

Given the extent of bone destruction and joint involvement, surgical management consisted of debridement and curettage of the lesion (Fig. 6)*,* followed by PMMA cement filling to restore structural stability (Fig. 7)*.* Due to the extension of the infectious process into the knee joint, a knee washout was also performed to evacuate intra-articular inflammatory debris and reduce bacterial load. In addition, the pre-tibial soft-tissue collection was surgically drained given its direct communication with the cortical breach.

**Fig. 8:**
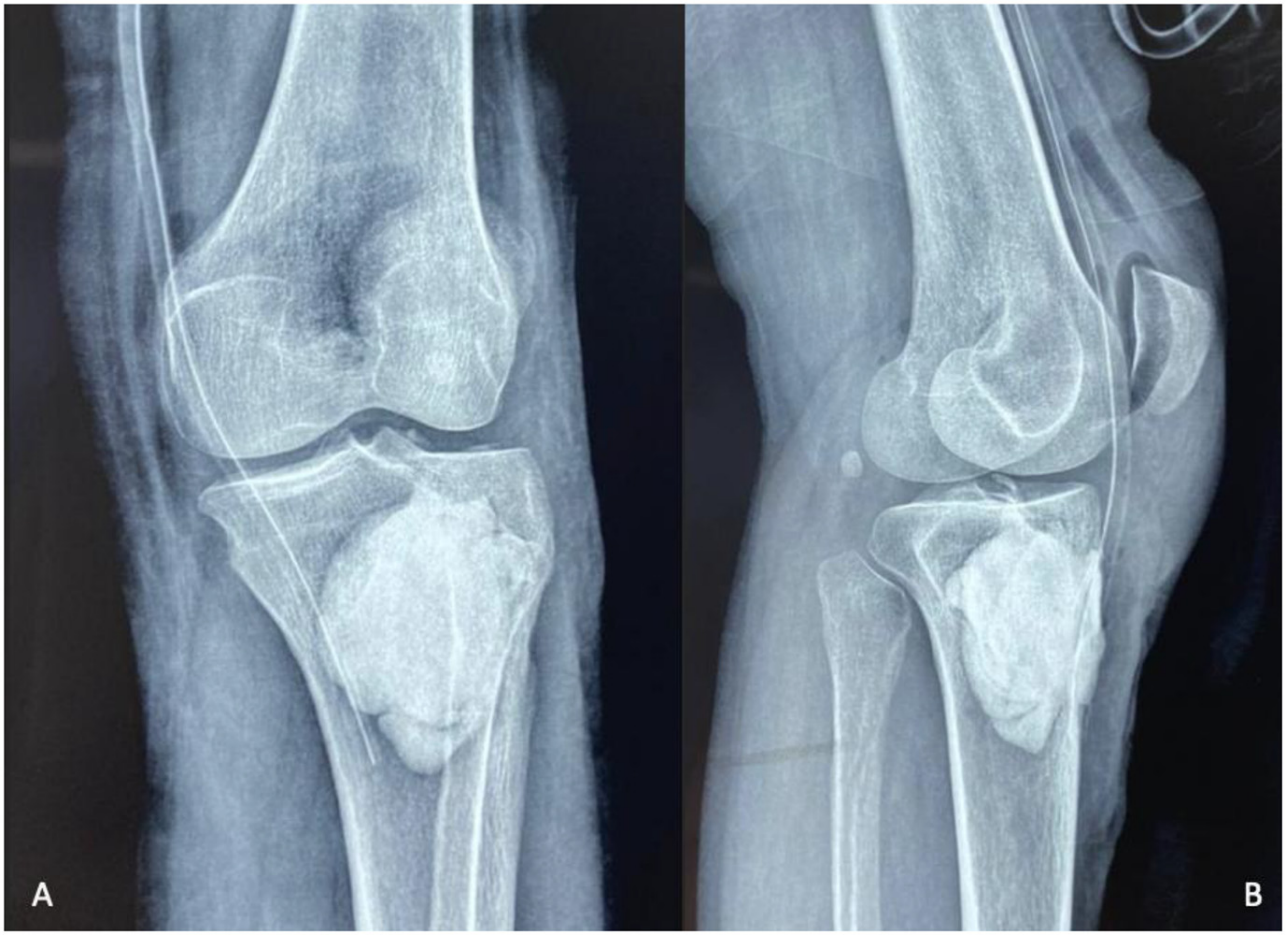
Postoperative control radiographs of the knee, anteroposterior (A) and lateral (B) views, showing the appearance after debridement and curettage of the lytic cavity, followed by filling with bone cement.

Histopathological examination confirmed granulomatous inflammation with epithelioid histiocytes, multinucleated giant cells, and caseous necrosis, while PCR testing identified *Mycobacterium tuberculosis*. Postoperative radiographs showed satisfactory filling of the defect (Fig. 8)*.* The patient was started on a standard 9-month antituberculous regimen (2RHZE/7RH) with early mobilization.

**Fig. 9:**
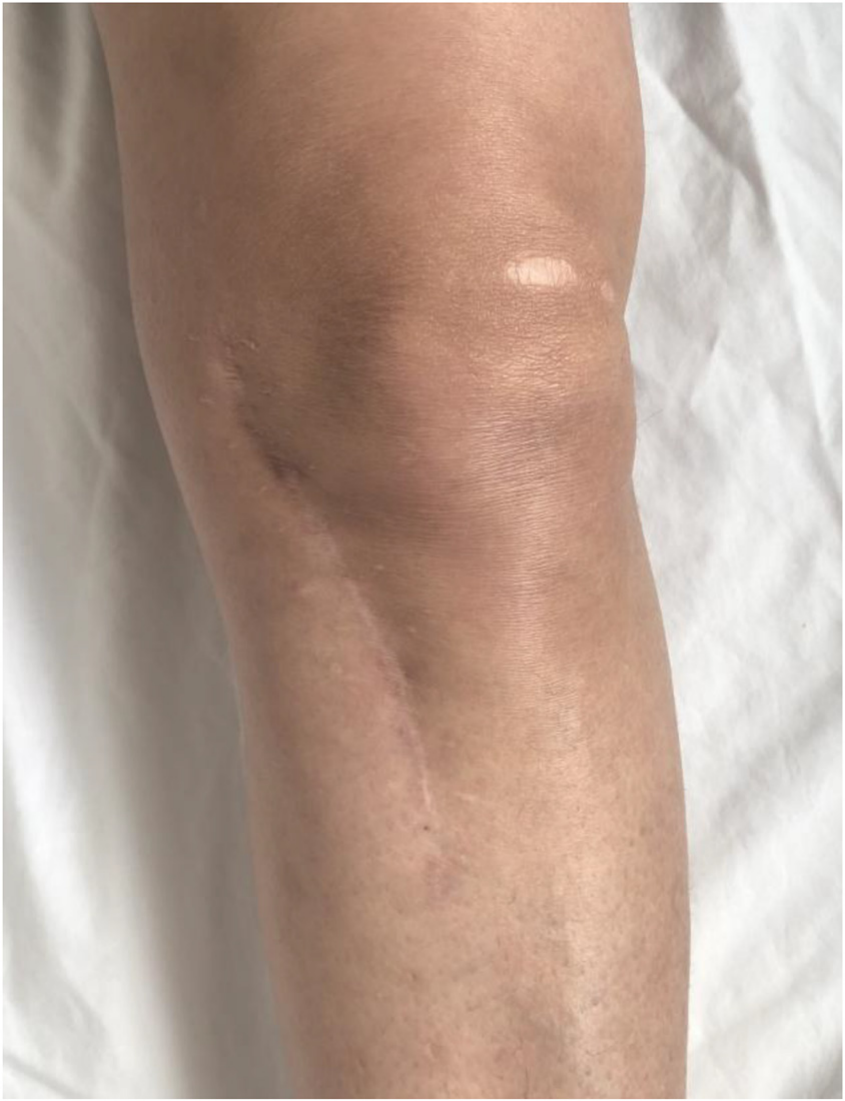
Clinical image at the last follow-up showing no knee swelling and a clean, well-healed surgical scar.

**Fig. 10:**
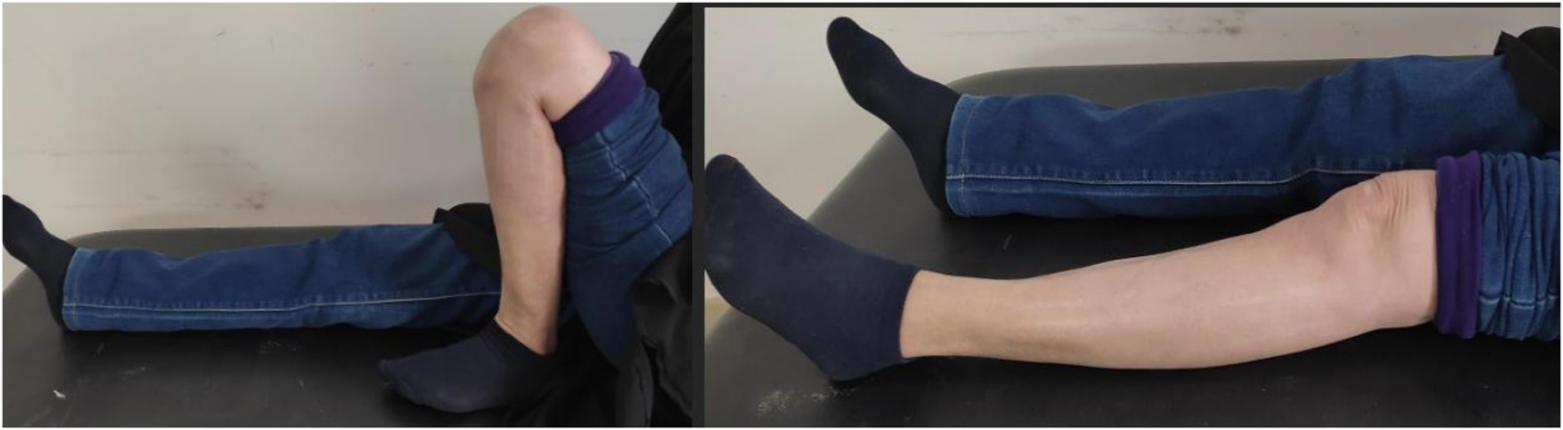
Clinical evaluation at the last follow-up shows full postoperative recovery of joint range of motion, with complete flexion and no flexion contracture.

**Fig. 11:**
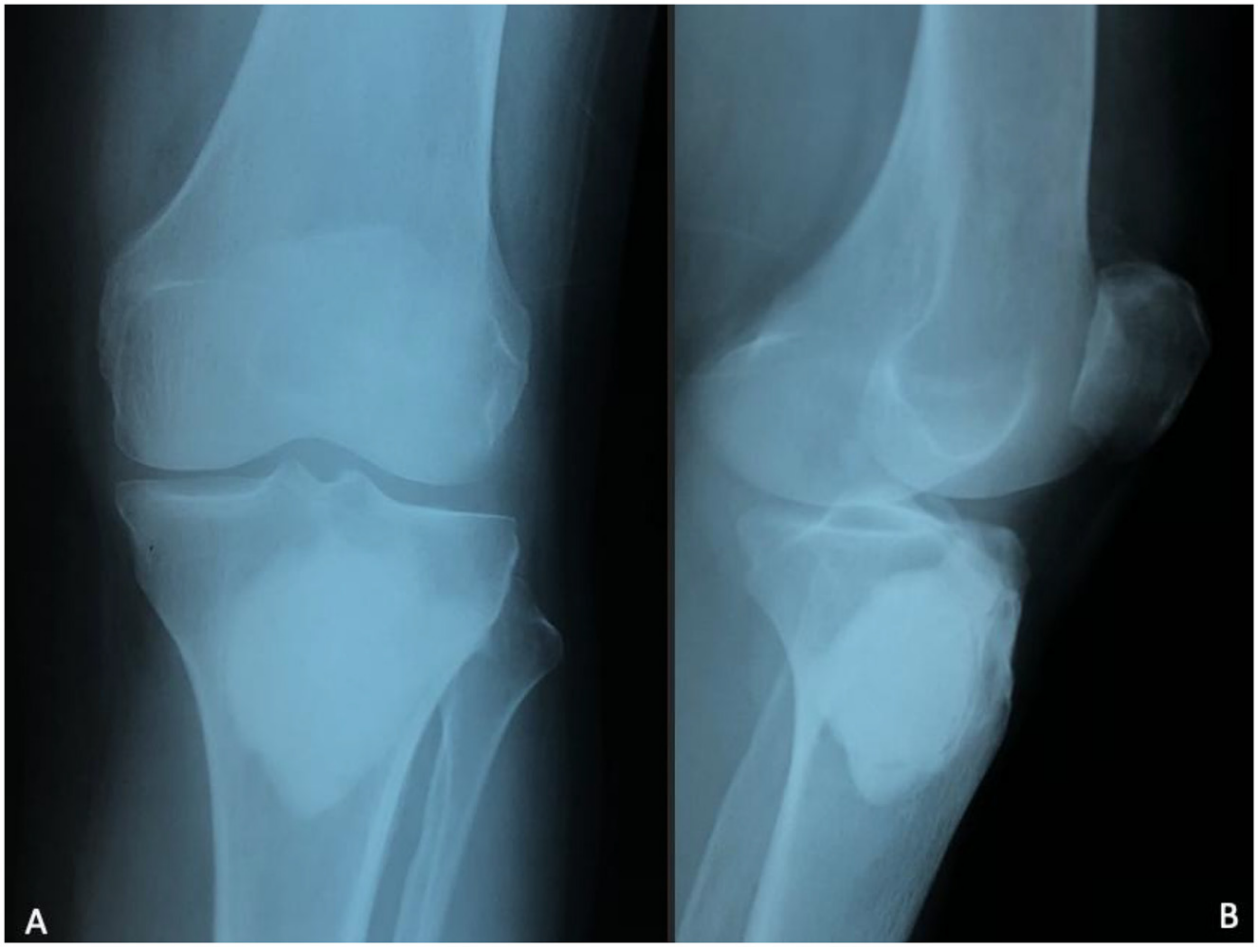
Follow-up radiographs at the last evaluation show no recurrence of the lytic process, with the bone cement in place at the proximal end of the tibia.

At final follow-up, she exhibited full knee range of motion (Fig. 10)*,* complete pain relief, and no residual swelling (Fig. 9), and radiographs confirmed the stability of the cement and absence of recurrence (Fig. 11)*.*

## Discussion

Tuberculous involvement of the musculoskeletal system represents a relatively uncommon manifestation of extrapulmonary tuberculosis, accounting for approximately 1%-3% of all tuberculosis cases [[1], [2], [3], [4]]. Although rare in high-income settings, it remains more prevalent in regions where tuberculosis is endemic and where access to early diagnostic resources may be limited [2,4,5]. The clinical and radiologic variability of skeletal tuberculosis often leads to delayed diagnosis—particularly when systemic manifestations are absent, as frequently observed in paucibacillary forms [2,[6], [7], [8]].

In the present case, the patient developed tuberculous osteomyelitis of the proximal tibia with secondary extension into the knee joint, ultimately presenting as a chronic monoarthritis. This progression underscores the well-recognized continuum between intraosseous tuberculosis and adjacent joint involvement, particularly when metaphyseal lesions breach the cortex and spread into synovial structures [1,5]. Thus, although the disease may originate in bone, the evolution toward osteoarticular involvement is common and should be anticipated clinically and radiologically.

The onset of tuberculous osteomyelitis is typically insidious and nonspecific. Most patients report localized pain, swelling, and progressive functional impairment—features that were also present in our case [2,6]. Constitutional symptoms classically associated with tuberculosis (fever, night sweats, weight loss) are often absent in musculoskeletal forms, contributing to diagnostic delay [2,6,8]. As reported in the literature, laboratory markers such as ESR and CRP may be normal or only moderately elevated; in our patient, both were raised, providing supportive but nonspecific evidence of inflammation [3,7].

Initial plain radiographs often lack sensitivity in early disease. When abnormalities do appear, bone tuberculosis typically produces lytic metaphyseal lesions with variable reactive sclerosis, depending on the stage of evolution [1,5]. The pattern may include cortical thinning, marginal erosions, or subtle periosteal reaction. Importantly, reactive sclerosis should be described as a response surrounding the lytic focus, rather than a coexisting sclerotic lesion. Mischaracterizing these changes as features seen in inflammatory arthropathies can be misleading, since classic inflammatory arthropathies do not cause lytic destruction with surrounding sclerosis [1,5].

In our case, CT provided excellent characterization of the lytic metaphyseal cavity with cortical breakthrough, thereby explaining the secondary intra-articular extension and subsequent knee arthritis [1,5]. CT also delineated the adjacent soft tissue changes and guided the surgical approach. MRI—although not used in our case—remains the most sensitive modality for early detection of marrow involvement, soft tissue extension, synovial inflammation, and early joint effusion, and is often recommended when radiographs and CT are inconclusive [1,9].

Definitive diagnosis rests on histopathological confirmation. The classical triad—epithelioid granulomas, Langhans-type giant cells, and central caseous necrosis—was clearly identified in our specimen [3]. Microbiological confirmation by PCR or culture further supports the diagnosis, especially in paucibacillary extrapulmonary forms [3,7,10].

The differential diagnosis of tuberculous osteomyelitis includes chronic pyogenic osteomyelitis, which may resemble to tuberculosis but usually shows more pronounced periosteal reaction and sequestration [5,11]. Several benign lytic bone lesions—such as unicameral bone cysts, enchondroma, osteoid osteoma, and giant-cell tumor—also mimic tuberculosis due to their well-defined metaphyseal lytic appearance [[11], [12], [13]]. In adults, primary bone tumors, lymphoma, and metastatic lesions must also be considered, especially when imaging shows aggressive cortical erosion or soft-tissue extension [[11], [12], [13], [14]]. Recent reviews highlight that tuberculosis can imitate both benign and malignant bone pathologies, reinforcing the need for biopsy with histopathology and mycobacterial testing to reach a definitive diagnosis, particularly in regions where tuberculosis remains endemic [2,4,5,11].

In the present case, surgical intervention was essential due to both cortical destruction of the proximal tibia and secondary extension of the infection into the knee joint. The patient underwent surgical debridement and curettage of the tibial osteolytic cavity, which allowed removal of necrotic tissue and reduction of bacterial load. The resulting bone defect was then filled with bone cement, providing immediate structural stability and facilitating radiologic surveillance for potential recurrence [2,3].

Given the intra-articular spread of the tuberculous process, we additionally performed a knee joint washout. This step was crucial to evacuate inflammatory debris and caseous material, reduce intra-articular bacterial burden, and limit the progression toward chronic synovitis or joint destruction. Joint irrigation is widely recommended in cases where osteomyelitis breaches the cortex and leads to secondary arthritis, as it improves local control of infection and enhances functional outcomes [2,15].

Postoperatively, the patient received a standard antituberculous regimen with a 2-month intensive phase (Rifampicin, Isoniazid, Pyrazinamide, Ethambutol) followed by a continuation phase with Rifampicin and Isoniazid, in accordance with international guidelines [2,3]. Clinical recovery was excellent, with full resolution of pain, restoration of knee mobility, and no evidence of recurrence on radiologic follow-up.

## Conclusion

This case highlights the diagnostic complexity of tuberculous osteomyelitis, particularly when it progresses to secondary joint involvement. The combination of slowly progressive symptoms, nonspecific laboratory findings, and variable radiologic appearances requires clinicians to maintain a high index of suspicion, especially in endemic regions. Early multimodal imaging and timely biopsy remain essential for accurate diagnosis and initiation of effective therapy. Ultimately, this report reinforces the importance of considering tuberculous osteomyelitis in the differential diagnosis of chronic bone lesions and monoarthritis, in order to prevent delayed management and irreversible joint damage.

## Authors' contributions

Ilyesse Haichour: Study concept, data collection, data analysis, writing paper.

Kamal Benalia: Study concept, data collection, data analysis, writing paper.

Najib Abdeljaouad: Supervision and data validation.

Hicham Yacoubi: Supervision and data validation.

## Patient consent

Written informed consent for publication of this case study was obtained from the patient.

## References

[1] Pattamapaspong N., Laohawiriyakamol T., Chaiwatanarat T., Churojana A., Chiewvit P., Wongsripuemtet J. (2023). Imaging of musculoskeletal tuberculosis. Br J Radiol.

[2] Marais L.C., Nieuwoudt L., Nansook A., Menon A., Benito N., Castelein R.M. (2023). Tuberculous arthritis of native joints: a systematic review and European Bone and Joint Infection Society workgroup report. J Bone Joint Infect.

[3] Panico C.T., de Oliveira P.R., Carvalho V.C., Leite M.S., Rosa B.B., Ferreira G. (2023). Clinical–epidemiological profile of confirmed cases of osteoarticular tuberculosis. J Bone Joint Infect.

[4] Souza AA, Alves RS, Pereira PF, Vasconcellos A, Lima LO, Barros R, et al. Manifestações osteoarticulares na tuberculose: uma revisão de literatura. *Educapes*. 2023–24. 10.1590/educapes.tuberculose.osteoarticular.2023

[5] El-Sharkawy M., Shalaby S., El-Adawy A., Nassif S., Abd El-Hamid S. (2024). Spectrum of imaging findings in osteoarticular tuberculosis: a pictorial review. Egypt J Radiol Nucl Med.

[6] Herdea A., Marie H., Negrila I.A., Balanescu R.N., Ionescu R., Vlad D.C. (2024). Reevaluating pediatric osteomyelitis with osteoarticular tuberculosis: addressing diagnostic delays and improving outcomes. Children (Basel).

[7] Gebrehana A.W., Munye G., Mekonen A.K., Fekadu S., Kassie G. (2024). Bilateral tuberculous dactylitis of both hands and feet in a toddler: a rare presentation of skeletal tuberculosis. BMC Infect Dis.

[8] Ramadugu R., Suvvari T.K., Ramadugu S., Sajja S. (2024). A rare case of osteoarticular tuberculosis and tuberculous osteomyelitis of the left foot without pulmonary involvement. Radiol Case Rep.

[9] Jadawala V.H., Deshpande S.V., Salwan A., Dhok A., Dhingra S., Yadav S. (2024). Tuberculous osteomyelitis of the calcaneum: a rare case. Cureus.

[10] Xu Q., Jing X., Zheng M., Zhong N., Liu Y., Fei W. (2024). Bone features reinforce differential diagnosis between tuberculous spondylitis and brucellosis spondylitis. BMC Infect Dis.

[11] Purwanto B.S., Bayusentono S., Martanto T.W., Zulkarnain A., Yazid H. (2024). Osteomyelitis tuberculosis in long bone mimicking bone malignancy: a case report. World J Adv Res Rev.

[12] Ulhaque F., Rahman S.H., Rai A., Singh V., Kumar R. (2024). Unifocal tubercular osteomyelitis of ulna diaphysis in a child: a case report. J Orthop Case Rep.

[13] Malihy Z., Benaissa E., Ben Lahlou Y., Maleb A., Elouennass M. (2023). Osteoarticular tuberculosis of the ankle, a rare localization: a case report. Access Microbiol.

[14] Wentao L., Shuxia X., Guoxing Z., Xiaoqin S., Fangyong L. (2024). Diagnosis of multiple tuberculous muscle abscesses in a patient with systemic lupus erythematosus by metagenomic next-generation sequencing: case report & literature review. BMC Infect Dis.

[15] Wang L., Zhang Y., Yang F., Liu S., Chen J. (2024). Diagnostic challenges of tuberculous osteomyelitis: a clinico-radiologic review. Infect Dis Rep.

